# Missed diagnoses: Clinically relevant lessons learned through medical mysteries solved by the Undiagnosed Diseases Network

**DOI:** 10.1002/mgg3.1397

**Published:** 2020-07-30

**Authors:** Heidi Cope, Rebecca Spillmann, Jill A. Rosenfeld, Elly Brokamp, Rebecca Signer, Kelly Schoch, Emily Glanton, Jennifer A. Sullivan, Ellen Macnamara, Sharyn Lincoln, Katie Golden‐Grant, Undiagnosed Diseases Network, James P. Orengo, Gary Clark, Lindsay C. Burrage, Jennifer E. Posey, Jaya Punetha, Amy Robertson, Joy Cogan, John A. Phillips, Julian Martinez‐Agosto, Vandana Shashi

**Affiliations:** ^1^ Division of Medical Genetics Department of Pediatrics Duke University Medical Center Durham NC USA; ^2^ Department of Molecular and Human Genetics Baylor College of Medicine Houston TX USA; ^3^ Division of Medical Genetics and Genomic Medicine Vanderbilt University Medical Center Nashville TN USA; ^4^ Department of Human Genetics University of California Los Angeles CA USA; ^5^ Department of Biomedical Informatics Harvard Medical School Boston MA USA; ^6^ Undiagnosed Diseases Program Common Fund Office of the Director National Institutes of Health NIH Bethesda MD USA; ^7^ Division of Genetics and Genomics Department of Pediatrics Boston Children’s Hospital Boston MA USA; ^8^ Division of Medical Genetics University of Washington Seattle WA USA; ^9^ Department of Neurology Baylor College of Medicine Houston TX USA

**Keywords:** exome sequencing, genome sequencing, phenotyping, targeted genetic testing, variant interpretation

## Abstract

**Background:**

Resources within the Undiagnosed Diseases Network (UDN), such as genome sequencing (GS) and model organisms aid in diagnosis and identification of new disease genes, but are currently difficult to access by clinical providers. While these resources do contribute to diagnoses in many cases, they are not always necessary to reach diagnostic resolution. The UDN experience has been that participants can also receive diagnoses through the thoughtful and customized application of approaches and resources that are readily available in clinical settings.

**Methods:**

The UDN Genetic Counseling and Testing Working Group collected case vignettes that illustrated how clinically available methods resulted in diagnoses. The case vignettes were classified into three themes; phenotypic considerations, selection of genetic testing, and evaluating exome/GS variants and data.

**Results:**

We present 12 participants that illustrate how clinical practices such as phenotype‐driven genomic investigations, consideration of variable expressivity, selecting the relevant tissue of interest for testing, utilizing updated testing platforms, and recognition of alternate transcript nomenclature resulted in diagnoses.

**Conclusion:**

These examples demonstrate that when a diagnosis is elusive, an iterative patient‐specific approach utilizing assessment options available to clinical providers may solve a portion of cases. However, this does require increased provider time commitment, a particular challenge in the current practice of genomics.

## 1 INTRODUCTION

The Undiagnosed Diseases Network (UDN) is a collaborative nationwide research study, funded by the National Institutes of Health Common Fund, tasked to solve the most challenging medical mysteries using team science and innovative approaches, including adopting advanced technologies (Gahl, Wise, & Ashley, 2015). Since 2015, the UDN has evaluated over 1,300 participants resulting in diagnoses for over 380 (~28% network‐wide diagnostic rate). Since more than 70% of rare diseases are thought to have a genetic basis (Nguengang Wakap et al., 2019), genomic sequencing is often an essential part of the UDN evaluation. Currently, more participants in the UDN receive genome sequencing (GS) than exome sequencing (ES), since many UDN participants (>70%, V.S., unpublished data) now enter the study with prior, non‐diagnostic ES. Few studies have systematically compared the diagnostic yield of GS and ES in the same patient population, but from available data it appears that GS has an approximate 9 to 16% improved diagnostic yield over ES (Alfares et al., 2018; Shashi et al., 2019). There have been calls to consider ES/GS as first‐tier tests in individuals with presumed genetic diseases (Bick, Jones, Taylor, Taft, & Belmont, 2019; Scocchia et al., 2019; Srivastava et al., 2019); however, ES/GS is not always necessary or sufficient to reach a diagnosis. Careful consideration of the phenotype in combination with utilization of appropriate first‐tier and targeted testing may reveal a diagnosis without necessitating ES/GS (Pena et al., 2018).

We present 12 participant examples that illustrate how the thoughtful use of readily available clinical methods and tools can result in diagnosis. All of these participants had prior non‐diagnostic evaluations by specialists locally, including a geneticist in all but three cases. While 7/12 participants received a diagnosis through ES/GS, GS was not necessary for any and ES was only necessary for 2/12; relevant targeted testing would have sufficed for 10/12. The lessons learned from these individuals are directly relevant to clinical practice as all of these participants could have been diagnosed utilizing clinically available methods.

## 2 METHODS

### 2.1 Ethical compliance

Informed consent had been obtained from each UDN participant or participant's parent/legal guardian to participate in the NIH‐UDN protocol (15‐HG‐0130). Individual 10 provided consent to participate in an ES research study at Duke (Pro00032301).

### 2.2 Participant selection

The UDN Genetic Counseling and Testing Working Group submitted case vignettes that illustrated how clinically available methods resulted in diagnoses. Vignettes for 16 participants were submitted for consideration and reviewed by HC and VS. Some participants illustrated the same clinical lesson; therefore, 11 participants from five UDN clinical sites (Duke, Baylor, Vanderbilt, UCLA and NIH) were included. Individual 10 was included to highlight a clinically important lesson that was not illustrated by any of the UDN participants. The case vignettes were classified into three themes following the logic of the lessons illustrated by each—phenotypic considerations, selection of genetic testing, and evaluating ES/GS variants and data (Table 1). Some participants demonstrated lessons that could fall under more than one theme, and in those instances, we selected the theme that best aligned. Utilizing the UDN diagnosis coding tool, each diagnosis was classified as either certain or highly likely (Splinter et al., 2018).

**Table 1:** Details of 12 participants that highlight clinically relevant diagnostic lessons

ID	Phenotype	Negative pre‐evaluation testing	Method diagnosis achieved	Diagnosis	Reason diagnosis initially missed	Lessons learned
Phenotypic considerations						
1	6‐year‐old female with severe global developmental delay, failure to thrive, hypotonia, refractory generalized epilepsy, regression of skills, and normal head circumference	Karyotype SNP CMA Fragile X syndrome Prader‐Willi/Angelman syndrome DNA methylation *SLC6A8* sequencing and del/dup	*MECP2* sequencing	Atypical Rett syndrome Heterozygous pathogenic variant in *MECP2* NM_004992.3:c.316C>T, p.R106W	Lack of recognition of the phenotypic spectrum of disease. *MECP2* testing was not ordered as patient did not have microcephaly and no period of normal development	When generating differential diagnoses, consider the phenotypic spectrum of a disorder due to variable expressivity
2	3‐year‐old female with severe global developmental delay, failure to thrive, dysmorphic features, hypotonia and hypo‐ and hyperpigmented skin lesions	SNP CMA Russell‐Silver syndrome DNA methylation Trio ES	Skin biopsy with karyotype of fibroblasts	Triploid/diploid mosaicism 69,XXX[7]/46,XX[13]	Prior testing performed on less sensitive sample type. Patient with phenotypic evidence of mosaicism had genetic testing on DNA from blood	When somatic mosaicism is suspected, consider starting with first‐tier genetic testing on skin or other affected tissue
3	20‐year‐old female with cognitive decline, status epilepticus at age 19, cerebral atrophy, small lactate peak on MRS, increased and abnormally shaped mitochondria on muscle biopsy	mtDNA genome sequencing Trio ES	mtDNA genome sequencing on DNA from muscle biopsy	Heteroplasmic mitochondrial disease Pathogenic variant in *MT‐TT* (m.15923A>G) at 21% heteroplasmy	Prior testing performed on less sensitive sample type. Patient with phenotypic evidence of mitochondrial disease had genetic testing on DNA from blood	If the phenotype is consistent with a mitochondrial disorder, consider genetic testing on muscle, other affected tissue or tissue with high mitochondria content, especially if testing on blood is nondiagnostic
Selection of genetic testing						
4	53‐year‐old female with progressive spasticity and gait disturbance. Affected mother, sister and two brothers	HSP panel (7 autosomal dominant genes) HSP panel (16 autosomal dominant genes)	ES *Could have been detected on X‐linked HSP panel	X‐linked adrenomyeloneuropathy Heterozygous likely pathogenic variant in *ABCD1* NM_000033.4:c.2035T>A, p.W679R	Prior testing ordered based on incorrect presumed inheritance pattern. Patient had two autosomal dominant HSP panels that did not include X‐linked genes	All inheritance patterns that are relevant to the individual should be considered when selecting genetic tests
5	17‐year‐old male with bilateral inguinal hernias, pectus excavatum, height at the 10−25th percentile, normal ophthalmology exam, aortic root size at the upper limits of normal and chronic kidney disease	*FBN1* sequencing and del/dup *TGFBR1/2* sequencing and del/dup Homocysteine	ES *Could have been detected on repeat *FBN1* sequencing	Marfan syndrome Heterozygous pathogenic variant in *FBN1* NM_000138.4:c.871G>T, p.E291X	Prior testing utilized outdated methodology. Prior testing in 1999 consisted of *FBN1* mRNA sequencing from cultured fibroblasts. Variants resulting in premature truncation could be missed by that technology due to nonsense‐mediated decay	Consider the methods and limitations of prior genetic testing, especially tests done several years ago, as they may need to be repeated with updated methods
6	27‐year‐old female with large ventricular septal defect, scoliosis, learning disabilities, autism spectrum disorder and white matter abnormalities suggestive of demyelination	Karyotype *FBN1* sequencing *TGFBR2* sequencing	SNP CMA *Not detected on ES	1q21.1 microduplication syndrome 1.7 Mb duplication at 1q21.1‐q21.2 AND Clinical diagnosis of multiple sclerosis	Lack of follow‐up to reevaluate genetic testing options over time. The patient had a single genetics evaluation in 2007. CMA was not available at that time and the participant was never referred back to genetics to consider additional testing	Periodic reevaluation of individuals is important to consider newly available genetic tests. CMA can detect large CNVs that can be missed by ES. A patient may have more than one diagnosis leading to a blended and complex phenotype
7	9‐year‐old female with severe global developmental delay, seizures, hypotonia and hip dysplasia	SNP CMA Trio ES	CN+SNP CMA *Not detected on GS	Wieacker‐Wolff syndrome 96 kb deletion at Xp11.2 spanning exon 1 of *ZC4H2*	Limitations of tests in detecting CNVs. SNP CMA, ES and GS were not able to detect the deletion	The sensitivity of CMAs in detecting CNVs can vary significantly between laboratories, due to the platform utilized and laboratory thresholds for reporting variants. ES, and to a lesser extent GS, may miss large CNVs and thus performing a CMA to detect these is important
8	60‐year‐old female with multiple benign neck paragangliomas. Extensive family history of paragangliomas	Hereditary pheochromocytoma and paraganglioma NGS panel	GS *Could have been detected on deletion/ duplication testing of *SDHD*	Paragangliomas 1 2.1 kb deletion at 11q23.1 encompassing exon 4 of *SDHD*	Limitations of gene panels in detecting CNVs. Patient had NGS panel which included *SDHD*, but did not include deletion/duplication testing	Sequencing panels alone cannot reliably detect CNVs, especially if they are NGS‐based. If panel sequencing is negative, subsequent deletion/duplication testing, such as MLPA/exon array should be considered
Evaluating ES/GS variants and data						
9	15‐year‐old female with mild motor delays, progressive muscle weakness and seizures	Cytochrome C oxidase deficiency Trio ES (non‐diagnostic but reported VUS in candidate gene *QRICH1*)	ES reanalysis	Ververi‐Brady syndrome De novo heterozygous pathogenic variant in *QRICH1* NM_017730.2:c.1378C>T, p.Q460X	Disease‐gene association not published at time of ES. *QRICH1* was not known to cause disease when ES was first completed, but commercial laboratory reported it as a candidate gene	ES reanalysis can result in diagnosis, however in some cases reinterpretation of a VUS by the clinical team that incorporates review of the interim literature can achieve diagnosis without requiring reanalysis
10	11‐year‐old female with congenital microcephaly, vertical nystagmus and profound intellectual disability	SNP CMA FISH for 22q11 deletion Angelman syndrome methylation analysis and *UBE3A* sequencing and del/dup *MECP2* sequencing and del/dup	ES	*KARS1‐*related spectrum disorder Compound heterozygous likely pathogenic variants in *KARS1* NM_001130090.1:c.169G>C, p.A57P and NM_001130090.1:c.1598C>G, p.P533F	Reference transcript nomenclature discrepancy *KARS1* reference transcript reported by the commercial lab for this patient (NM_001130090.1) was different from transcript nomenclature in the relevant literature (NM_005548.2)	Providers should be cognizant of differences in reference transcripts used for variant nomenclature when comparing variants in the literature
11	32‐year‐old male with chronic bronchiectasis, progressive emphysema and abnormal salivary gland morphology. Similarly affected daughter and sister	Research trio ES	GS with manual interrogation of *FBN1* due to phenotypic suspicion *Could have been detected on *FBN1* sequencing or ES	Marfan syndrome Heterozygous pathogenic variant in *FBN1* NM_000138.4:c.3712G>A, p.D1238N	Commercial lab did not report the variant Phenotype information provided to the lab was not consistent with Marfan syndrome. Clinical laboratory detected, but did not report, the *FBN1* variant due phenotypic filtering	For optimal variant filtering, it is important to provide accurate and detailed phenotypic information to testing laboratories, including medical manifestations that may be unrelated to the reason for testing. If a particular diagnosis is suspected, but ES/GS is negative, clinicians should discuss the phenotype with the testing laboratory and ask the lab to manually inspect the raw data for specific genes of interest
12	9‐year‐old male with refractory epilepsy, developmental delay and autistic features	Karyotype SNP CMA Fragile X syndrome Atypical Rett syndrome panel Trio ES	GS *Could have been detected on ID/autism/epilepsy panel or repeat ES but not on ES reanalysis	Intellectual disability, autosomal dominant 5 Heterozygous pathogenic variant in *SYNGAP1* NM_006772.2:c.3415insA, p.T1140DfsX13	Commercial lab did not detect the variant ES capture kit used by the commercial laboratory in 2016 only covered 56% of *SYNGAP1*	Pathogenic coding variants can be missed on ES due to exome capture limitations. ES reanalysis is limited to variants captured and sequenced by the ES platform utilized at that time

Abbreviations: CMA, chromosomal microarray; CNV, copy number variant; ES, exome sequencing; GS, genome sequencing; HSP, hereditary spastic paraplegia; mtDNA, mitochondrial DNA; NGS, next generation sequencing; VUS, variant of uncertain significance.

## 3 RESULTS

### 3.1 Phenotypic considerations

#### 3.1.1 Participant 1

A 6‐year‐old female was evaluated due to severe global developmental delays, failure to thrive, feeding difficulties, hypotonia and refractory generalized epilepsy, all occurring in the first two years of life. At one year of age, she developed stereotypical movements of bringing her hands to her mouth and lost the ability to grasp toys and food with her hands. The head circumference and brain MRIs were normal. Pre‐UDN genetic testing was negative (Table 1).

In the UDN, the participant's head circumference was at the 63rd percentile. Features suggestive of Rett syndrome were noted, including breathing disturbances; bruxism; small, cold feet and diminished response to pain, in addition to the previously recognized hypotonia, hand stereotypies and history of regression. Criteria for atypical Rett syndrome were fulfilled and *MECP2* (MIM# 300005) gene sequencing revealed a known heterozygous pathogenic variant (c.316C>T, p.R106W), which accounts for ~4% of pathogenic variants in *MECP2* (Neul et al., 2010; Webb & Latif, 2001). The mother was negative for the variant and while the father was not available, it was presumed that the variant was de novo. On review of the participant's medical records, the local health care team had considered Rett syndrome, but testing had not been pursued, since she did not have microcephaly and there was no period of normal neurodevelopment. Careful consideration of the phenotypic spectrum seen in Rett syndrome led to *MECP2* sequencing and a certain diagnosis of atypical Rett syndrome (MIM# 312750).

##### Lesson learned

When generating differential diagnoses, consider the phenotypic spectrum of a disorder due to variable expressivity.

#### 3.1.2 Participant 2

A 3‐year‐old female was evaluated due to being small for gestational age, severe global developmental delays, failure to thrive, dysmorphic features, hypo‐ and hyperpigmented skin lesions in a segmental pattern on the bilateral lower extremities and hypotonia. Pre‐UDN genetic testing on blood was nondiagnostic (Table 1).

On UDN evaluation, height and weight were well below the 1st percentile (−8.06 and −7.9 *SD*, respectively) and head circumference was at the 4th percentile. Skin biopsies of the hypo‐ and hyperpigmented lesions were obtained and the derived fibroblasts were sent for chromosome analysis. This resulted in a certain diagnosis of diploid/triploid mosaicism (mixoploidy), with a karyotype of 69,XXX[7]/46,XX[13]. The participant's severe growth failure, skin lesions, and severe developmental delays were highly consistent with mosaic triploidy of maternal origin (Carson et al., 2018). The referring geneticist had considered this diagnosis, but did not proceed with the skin biopsy, as repeat ES of DNA from fibroblasts was denied by insurance.

##### Lesson learned

When somatic mosaicism is suspected, consider starting with first‐tier genetic testing on skin or other affected tissue.

#### 3.1.3 Participant 3

A 20‐year‐old female presented with a 4‐year history of gradual cognitive decline (IQ decreased from 101 to 77) and status epilepticus at age 19. Pre‐UDN testing included a brain MRI 1‐month after the status epilepticus, demonstrating extensive cerebral atrophy with small infarcts in the temporal and frontal lobes, and MRS, which showed a small lactate peak in the left basal ganglia. She underwent a brain biopsy, which showed microglial activation, reactive gliosis, and focal loss of myelin in the white matter. A muscle biopsy was obtained from the temporal muscle, which showed increased and abnormally shaped mitochondria. Genetic testing including mitochondrial (mtDNA) GS on blood was nondiagnostic (Table 1).

In the UDN, an ERG showed profound damage to both the photoreceptor and the inner retinal layers, consistent with a retinal dystrophy. Since the aggregate phenotype was highly suggestive of mitochondrial disease, mtDNA GS was performed on the pre‐UDN muscle biopsy sample, and a pathogenic variant was reported in *MT‐TT* (m.15923A>G) at 21.6% heteroplasmy, consistent with a certain diagnosis of *MT‐TT* (MIM# 590090) related mitochondrial disease. This variant had been reported in other affected individuals and was mostly undetectable in blood samples (Karppa, Kytovuori, Saari, & Majamaa, 2018). Repeat mtDNA GS with a new blood sample found the variant at 3.4% heteroplasmy. The participant's unaffected mother was not tested.

##### Lesson learned

If the phenotype is consistent with a mitochondrial disorder, consider genetic testing on muscle, other affected tissue or tissue with high mitochondria content, especially if testing on blood is nondiagnostic.

### 3.2 Selection of Genetic Testing

#### 3.2.1 Participant 4

A 53‐year‐old female was evaluated due to eight years of progressive spasticity and gait disturbance. Her mother and two brothers had onset of these symptoms in their 20s‐30s, and her sister was more mildly affected. Hereditary spastic paraplegia (HSP), likely autosomal dominant (AD), had been considered. A brain MRI and electromyogram/nerve conduction studies were normal. Pre‐UDN genetic testing consisting of two AD HSP panels was negative (Table 1).

The clinical evaluation through the UDN noted impaired vibration and pain sensation, hyperreflexia, and spasticity in the lower limbs, consistent with HSP. ES on the proband and all four affected family members found a shared likely pathogenic missense variant in *ABCD1* (MIM# 300371; c.2035T>A, p.W679R), resulting in a certain diagnosis of X‐linked adrenomyeloneuropathy (MIM#300100) (Korenke et al., 1998). Subsequent very long chain fatty acid testing was consistent with this diagnosis. Since spastic paraplegia can be a manifestation of this disorder, *ABCD1* is included in HSP panel tests, but only in those that include X‐linked HSP genes. The diagnosis had been missed because the participant's prior genetic testing only included AD HSP gene panels.

##### Lesson learned

All inheritance patterns that are relevant to the individual should be considered when selecting genetic tests.

#### 3.2.2 Participant 5

A 17‐year‐old male had skeletal features suggestive of a connective tissue disorder and chronic kidney disease (CKD). At age 19 months he had been evaluated for Marfan syndrome with bilateral inguinal hernias and mild aortic root dilatation. Genetic testing including *FBN1* (MIM# 134797) sequencing was negative (Table 1).

At the time of his UDN evaluation, his height was at the 10‐25th percentile, pectus excavatum was noted, ophthalmology exam was normal, aortic root size was at the upper limits of normal and he had developed nephrocalcinosis and CKD stage 3. Trio ES through the UDN found a pathogenic, de novo, heterozygous nonsense variant in *FBN1* (c.871G>T, p.E219X) resulting in a certain diagnosis of Marfan syndrome (MIM# 154700). The prior testing in 1999 consisted of *FBN1* mRNA sequencing from cultured fibroblasts, standard practice at the time. The truncated mRNA transcript due to the nonsense variant was likely destroyed by nonsense‐mediated decay, preventing RT‐PCR capture for sequencing, resulting in sequencing of only the normal allele. The performing laboratory had noted on the report that the participant was homozygous for all known polymorphic SNPs in the cDNA, which could have indicated that only one allele had been sequenced. Single gene sequencing of *FBN1* utilizing DNA would have detected the variant; however, this was not pursued by the UDN because of the CKD, for which ES was a better option. The participant's history of CKD was not explained by the Marfan syndrome diagnosis but could perhaps account for his atypical short stature.

##### Lessons learned

Consider the methods and limitations of prior genetic testing, especially tests done several years ago, as they may need to be repeated with updated methods.

#### 3.2.3 Participant 6

A 27‐year‐old female was evaluated due to a large ventricular septal defect, scoliosis, learning disabilities, attention deficit disorder, chronic fatigue syndrome and demyelination on brain MRI. In 2007, at age 17, she was evaluated by a geneticist and genetic testing was normal (Table 1). She was not evaluated by genetics again. At age 21 years, her local neurologists considered a diagnosis of multiple sclerosis due to fatigue and the demyelination. She also had developed anxiety and depression.

In the UDN, she was determined to have autism spectrum disorder. Repeat brain MRI showed a new non‐enhancing FLAIR hyperintense lesion within the periventricular white matter adjacent to the right occipital horn. Genetic testing included a CMA (not available in 2007), and trio ES. A 1.7 Mb interstitial duplication at 1q21.1‐1q21.2 was identified on the CMA, consistent with a certain diagnosis of 1q21.1 duplication syndrome (MIM# 612475). This diagnosis was thought to explain the participant's congenital heart disease, learning disabilities and perhaps the neuropsychiatric diagnoses. ES was nondiagnostic and failed to detect the 1q21.1 duplication. The history and brain MRI findings were determined to be consistent with a clinical diagnosis of multiple sclerosis by the UDN neurologist, and the mild disease course was attributed to medications. Multiple sclerosis is not currently known to be part of the 1q21.1 duplication syndrome phenotype.

##### Lessons learned

(a) Periodic reevaluation of individuals is important to consider newly available genetic tests. (b) CMA can detect large copy number variants (CNVs) that can be missed by ES. (c) A patient may have more than one diagnosis leading to a blended and complex phenotype.

#### 3.2.4 Participant 7

A 9‐year‐old female was evaluated with severe global developmental delays, seizures, hypotonia, and hip dysplasia. Pre‐UDN genetic testing including a SNP microarray in 2016 was nondiagnostic (Table 1).

Clinical evaluation in the UDN demonstrated upslanted palpebral fissures, low set ears, micrognathia, generalized hypotonia, profound lower limb muscle weakness, and intellectual disability. GS and a high‐density CMA with 2 million more probes than the prior CMA [oligonucleotide and SNP probes (CN+SNP array)], were performed (Hensel et al., 2017). The CN+SNP array revealed a 95 kb deletion at Xq11.2 spanning exon 1 of the *ZC4H2* gene (MIM# 300897), resulting in a certain diagnosis of X‐linked Wieacker‐Wolff syndrome (MIM# 314580). Parental testing indicated that it was de novo in the participant. “Carrier” females, while typically mildly affected, can have severe phenotypic features of Wieacker‐Wolff, including skeletal abnormalities such as hip dislocation and intellectual disability (Zanzottera et al., 2017).

Genome sequencing utilizing Manta for CNV calling was non‐diagnostic and did not detect the 95 kb deletion, and communication with the UDN core laboratory did not clarify why the CNV was not detected (Chen et al., 2016). We were also unable to obtain clarity from the commercial lab that performed the 2016 SNP microarray as to why the 95 kb deletion had not been reported. We surmise that the deletion would have been detectable, but may not have met the laboratory's reporting criteria due to its size and because it was not a well‐known microdeletion.

##### Lesson learned

(a) The sensitivity of CMAs in detecting CNVs can vary significantly between laboratories, due to the platform utilized and laboratory thresholds for reporting variants. (b) ES, and to a lesser extent GS, may miss large CNVs and thus performing a CMA to detect these is important.

#### 3.2.5 Participant 8

A 60‐year‐old female was evaluated due to multiple benign neck paragangliomas. Several extended family members were similarly affected, including two sisters, her father, and paternal aunts, uncles, and cousins. Pre‐UDN genetic testing consisted of a normal hereditary pheochromocytoma and paraganglioma (HPP) next generation sequencing (NGS) panel (Table 1).

Clinical evaluation of the proband in the UDN noted carotid and vagal paragangliomas, episodic hypertension with tachycardia and vocal cord paralysis. UDN GS on the proband and two of her affected family members detected a heterozygous 2.17 kb deletion within the *SDHD* gene (MIM# 602690). Subsequent arrayCGH of *SDHD* was pursued and confirmed a deleterious ~2.13 kb deletion encompassing exon 4 of *SDHD,* resulting in a certain diagnosis of paragangliomas 1 (MIM# 168000). While *SDHD* was included in the prior HPP NGS panel, the panel did not include deletion/duplication testing. Deletions in *SDHD* are a known cause of HPP, accounting for as much as 10% of pathogenic variants (Hoekstra et al., 2017).

##### Lessoned learned

Sequencing panels alone cannot reliably detect CNVs, especially if they are NGS‐based. If panel sequencing is negative, subsequent deletion/duplication testing, such as MLPA/exon array should be considered.

### 3.3 Evaluating ES/GS Variants and Data

#### 3.3.1 Participant 9

A 15‐year‐old female was evaluated due to mild motor delays, progressive muscle weakness and seizures. Pre‐UDN genetic testing included nondiagnostic trio ES in 2016, with a variant of uncertain significance (VUS) in a candidate gene *QRICH1* (MIM# 617387; Table 1).

At the time of her UDN evaluation, she was in the 9th grade, performing at a 4th‐6th grade level. The physical exam was significant for ptosis, mild myopathic facies, and decreased muscle mass, strength and reflexes in the lower extremities. Simultaneously, reanalysis of the trio ES data in a parallel study detected a novel de novo nonsense variant in *QRICH1* (c.1378C>T, p.Q460X). By this time, truncating variants in *QRICH1* had been published in AD Ververi‐Brady syndrome (Ververi, Splitt, Dean, Study, & Brady, 2018). The reported individuals had cognitive impairments, and one had muscle fatigue and weakness. This publication was shared with the commercial laboratory that had performed the pre‐UDN ES, resulting in reclassification of this novel variant in *QRICH1* as pathogenic and a certain diagnosis of Ververi‐Brady syndrome (MIM# 617982). Reinterpretation of the VUS by the local clinical team in light of the publication would have solved the case. However, time‐constrained clinicians would have difficulty in identifying interim new publications when undiagnosed patients may be seen only every few years.

##### Lessons learned

ES reanalysis can result in diagnosis, however, in some cases reinterpretation of a VUS by the clinical team that incorporates review of the interim literature can achieve diagnosis without requiring reanalysis.

#### 3.3.2 Participant 10

An 11‐year‐old female was evaluated by a Duke genomic sequencing study due to congenital microcephaly, vertical nystagmus, and profound intellectual disability. Prior genetic testing was normal (Table 1).

Trio ES detected compound heterozygous, likely pathogenic variants in *KARS1* (MIM# 601421). *KARS1* had recently been associated with neurological features that overlap our participant's (McMillan et al., 2015). The variants were Sanger confirmed in a CLIA‐certified laboratory and reported as c.169G>C, p.Ala57Pro and c.1598C>G, p.Pro533Arg (NM_001130089.1). The c.169G>C variant had been previously reported in the literature as c.85G>C, p.Ala29Pro using a different transcript (NM_005548.2) (Joshi et al., 2016). Applying this NM_005548.2 transcript, the second variant in our participant, c.1598C>G, would be c.1514C>G, p.Pro505Arg. Since another variant impacting the same codon c.1513C>T, p.Pro505Ser was reported in the *KARS1* literature, the evidence of pathogenicity of the variant in our participant was strengthened (Zhou et al., 2017). Thus, without careful attention to the variant annotation differences, the prior associations to the *KARS1*‐related neurological phenotypes may have been overlooked. The participant was given a likely diagnosis of *KARS1*‐related disorder.

##### Lesson learned

Providers should be cognizant of differences in reference transcripts used for variant nomenclature when comparing variants in the literature.

#### 3.3.3 Participant 11

A 32‐year‐old male was evaluated due to chronic bronchiectasis, progressive emphysema, abnormal salivary gland morphology, xerostomia, and carious teeth. His 3‐year‐old daughter was similarly affected, and his sister had died from childhood respiratory failure. Pre‐UDN genetic testing through a rare lung disease research program included research trio ES, which was nondiagnostic (Table 1).

UDN GS was performed prior to his clinical evaluation on the proband, his parents and his daughter. Results were nondiagnostic. During the UDN evaluation it was noted that the proband was tall, with arachnodactyly, inguinal hernia and aortic root aneurysm. His father had bilateral ectopia lentis and tall stature. The paternal grandmother and her brother also had bilateral ectopia lentis. Thus, Marfan syndrome was considered, and a UDN bioinformatician manually inspected the *FBN1* gene in the GS data. A known pathogenic variant in *FBN1* (c.3712G>A, p.Asp1238Asn) was detected and a certain diagnosis of Marfan syndrome (MIM# 154700) was given. The proband's father was positive for this variant and his daughter was negative. In discussions with the UDN core laboratory that performed the GS, this variant had been filtered out due to phenotypic mismatch. While the proband's symptoms that prompted referral remained undiagnosed, the diagnosis of Marfan syndrome was enabled by phenotype‐driven re‐examination of the GS data.

##### Lessons learned

(a) For optimal variant filtering, it is important to provide accurate and detailed phenotypic information to testing laboratories, including medical manifestations that may be unrelated to the reason for testing. (b) If a particular diagnosis is suspected, but ES/GS is negative, clinicians should discuss the phenotype with the testing laboratory and ask the lab to manually inspect the raw data for specific genes of interest.

#### 3.3.4 Participant 12

A 9‐year‐old male was evaluated due to refractory, early‐onset absence epilepsy, developmental delay, and autistic features. Pre‐UDN workup including trio ES in 2016 was non‐diagnostic (Table 1).

In the UDN, EEG demonstrated interictal generalized and localized epileptiform discharges, including absences. Quad GS with an unaffected sibling revealed a de novo, exonic, pathogenic variant (c.3415insA, p.T1140DfsX13) in *SYNGAP1* (MIM# 603384), resulting in a certain diagnosis of Intellectual disability, AD 5 (MIM# 612621), which also includes epilepsy. Upon inquiry, the commercial laboratory that had performed the trio ES reported that the exome capture kit used by the laboratory in 2016 only covered 56% of *SYNGAP1*. GS, which provides more uniform coverage of coding regions, resulted in a certain diagnosis for the participant. In this instance, reanalysis of the pre‐UDN ES data would not have solved this case, as the variant was not captured.

##### Lessons learned

(a) Pathogenic coding variants can be missed on ES due to exome capture limitations. (b) ES reanalysis is limited to variants captured and sequenced by the ES platform utilized at that time.

## 4 DISCUSSION

The UDN evaluates individuals with refractory complex medical conditions, some of whom have been seeking answers for decades (Spillmann et al., 2017; Splinter et al., 2018). Participants, such as the ones described here, have received diagnoses through approaches that are tractable clinically, such as reconsidering the phenotype, selecting optimal tests and reanalyzing/reinterpreting ES/GS data. Indeed, we have reported that 22% of UDN diagnoses were made due to clinical review and directed clinical testing (Splinter et al., 2018). Notably, 9/12 participants in this study had been evaluated by a genetics provider prior to participation. Each of the three broad thematic approaches that led to diagnoses would be tractable in clinical genetics practice. However, the current time constraints in clinical genetics practice pose a barrier to conducting the customized reiterative clinical and molecular reassessments that occurred in the 12 participants, especially since most of these activities would have to occur outside of the face‐to‐face reimbursable time (Attard, Carmany, & Trepanier, 2019; Fennell, Hunter, & Corboy, 2020; Maiese, Keehn, Lyon, Flannery, & Watson, 2019). We discuss the salient lessons within the three thematic approaches below.

### 4.1 Phenotypic considerations

#### 4.1.1 Customized phenotyping

UDN participants undergo personalized and multidisciplinary deep phenotyping during a multiday evaluation to aid in diagnosis (Ramoni et al., 2017). The UDN advantage of being able to coordinate all the evaluations in a short time span is difficult to achieve in clinical settings. In our cohort of 12 participants, thoughtful phenotyping played a significant role in diagnosis for five. These diagnoses were achieved through phenotype‐guided targeted testing utilizing relevant sample types (Participants 1, 2, 3), phenotype‐driven re‐examination of genomic data (Participant 11), and phenotype‐driven clinical diagnosis (Participant 6). Reconsidering a patient's phenotype in the context of the extant literature is vital in recognizing variable expressivity and then selecting the optimal tests. However, the time required can be a significant limiting factor for providers in a clinical setting.

An individual's phenotype is not always due to a single etiology, as evidenced by Participant 6 and the estimated 2%–7% of individuals undergoing ES who receive multiple genetic diagnoses (Posey et al., 2016; Smith et al., 2019). Individuals with multiple diagnoses have been found to have only slightly more organ systems affected (average of 4.3) than individuals with single diagnoses (average of 3.9). Furthermore, with dual diagnoses, half of the phenotypes are completely separate, and about half are completely, or partially, overlapping (Smith et al., 2019). Thus, it can be difficult to discern if the phenotype is indicative of more than one diagnosis. In this study, Participants 5 and 11 likely also have two conditions, with the second still unresolved.

#### 4.1.2 Mosaicism and heteroplasmy

Many genetic conditions have been observed in a mosaic state and are hard to diagnose (Cao et al., 2019). However, there are certain phenotypic clues such as segmental overgrowth and characteristic cutaneous patterns or lesions that are highly suggestive of somatic mosaicism (Kinsler et al., 2020). In the case of Participant 2, skin lesions suggestive of mosaicism were present, yet pre‐UDN testing was completed on DNA from blood instead of DNA from a biopsy of the affected tissue, which often has greater sensitivity for the detection of a mosaic variant (Mirzaa et al., 2016). Additionally, specific testing for mosaic conditions should be based on the differential diagnosis; reflexively ordering ES on skin fibroblasts because the patient was undiagnosed may have resulted in the triploidy remaining undetected since ES is not a reliable method to detect mosaic polyploidy (Posey, 2019). One practical limitation is that not all genetics clinics have access to laboratories that perform tissue culture.

Nearly all pathogenic variants in the mtDNA genome are heteroplasmic (Santibanez‐Koref et al., 2019). NGS is the gold standard for mtDNA GS and allows detection of low‐level heteroplasmy. In Participant 3, mtDNA GS on muscle was necessary to identify the heteroplasmic variant that was missed in blood. Skeletal muscle or liver tissues are ideal for mtDNA GS given their high mtDNA content and if available should be utilized as the primary sample for testing or if initial testing on blood is negative (Parikh et al., 2015).

### 4.2 Selection of genetic testing

#### 4.2.1 Evaluating prior testing

For undiagnosed individuals who underwent genetic testing many years previously, it is important to consider the limitations of prior genetic testing. Illustrating this is Participant 5, who underwent testing when the large size of *FBN1* (235 kb) precluded Sanger sequencing of genomic DNA and thus the more manageable 10 kb cDNA sequencing on fibroblasts was the standard (Collod‐Beroud & Boileau, 2002), but did not detect the nonsense variant. It is inevitable that testing methodologies will improve over the course of a lengthy diagnostic odyssey. For example, CMA technology has improved from the original BAC arrays that had a resolution of approximately 1 Mb (Ballif et al., 2008), to current CN+SNP microarrays that can reliably detect CNVs>100 kb (Mason‐Suares et al., 2013). For Participant 6, CMA was not obtained in 2007, as it was not recommended as a first‐tier genetic test until 2010 (Manning, Hudgins, Professional, & Guidelines, 2010; Miller et al., 2010). Repeat evaluation by genetics after 2010 would most likely have resulted in CMA testing and diagnosis.

Finally, Participant 4 reflects the need for consideration of modes of inheritance for the disease phenotype when selecting a gene panel. If X‐linked disease had been suspected, due to two male siblings who were more severely affected, the diagnosis may have been made without ES. Furthermore, the case highlights the importance of retrospectively reviewing the genes included in a panel before determining that a group of disorders has been comprehensively tested for.

#### 4.2.2 CNV detection

CN+SNP microarrays can detect some smaller CNVs ~10 kb in size within clinically relevant regions of the genome (Mason‐Suares et al., 2013). However, it is important to remember that detecting CNVs not only varies by CMA platform (type and number of probes included) but that criteria for reporting variants differ between commercial laboratories. Participant 7 illustrates both points (Table 2). While a CMA in 2016 was negative, in 2017, a CN+SNP array with more probes in a laboratory with a reporting threshold >50 kb detected the 96kb deletion (Hensel et al., 2017). However, some cytogenetics laboratories still do not routinely report deletions <300 kb (unless they are known microdeletions). Hence, it is important to consider laboratory thresholds as well as probe sensitivity for CMA selection.

**Table 2:** CNVs detected in three UDN participants that had been missed previously

Participant	CNV	Detected by	Not detected by
Participant 6	1.7 Mb duplication at 1q21.1‐q21.2	CN + SNP CMA	ES
Participant 7	96 kb deletion at Xp11.2	CN + SNP CMA	SNP CMA, ES, GS
Participant 8	2.13 kb deletion	GS	NGS panel

Abbreviations: CNV, copy number variant; ES, exome sequencing; GS, genome sequencing; NGS, next generation sequencing; UDN, Undiagnosed Diseases Network.

Numerous algorithms for calling CNVs from ES/GS data have been developed, but validation is lacking, leading to immense variability between laboratories (Tattini, D'Aurizio, & Magi, 2015). The short nature of reads (~150 bp) with currently used NGS methods, variable sequencing depths in ES and the large number of benign CNVs in GS data present challenges for the detection and annotation of these. The estimated sensitivity for CNV detection utilizing ES‐based CNV callers is ~76% in comparison to CMA (Krumm et al., 2012). In this study, ES was not able to detect the 1.7 Mb duplication in Participant 6, nor the 96 kb deletion in Participant 7, which were both detected by CMA (Table 2). There have been recent calls for ES to replace CMA as the first genetic test in individuals with neurodevelopmental disorders (Srivastava et al., 2019); however, it is important to recognize the limitations of ES in detecting CNVs, even if laboratories run a SNP array in conjunction with the ES and may be able to detect some CNVs.

GS has improved sensitivity over ES and CMA in detecting smaller CNVs <50 kb (Gross et al., 2019). In Participant 8, a 2.13 kb deletion in *SDHD* that would not have been detectable by CMA was detected by GS (Table 2). Yet there are still limitations to detecting CNV in GS data, as illustrated by the inability of GS to detect the 96 kb deletion in Participant 7. In summary, CNVs are a significant contribution to genetic disease (5%–35%) that remain a challenge to detect and characterize (Truty et al., 2019). While there is no one method that is 100% sensitive in detecting CNVs, a combination of approaches may result in increased diagnostic yield.

### 4.3 Evaluating ES/GS variants and data

#### 4.3.1 Transcripts

Accurately and unambiguously describing a variant and its location is a critical part of genomic sequencing. Two factors that complicate this process are the presence of multiple human genome annotation databases (e.g. RefGene, Ensembl, UCSC Genome Browser) and the observation that most genes in the human genome have multiple isoforms (Pan, Shai, Lee, Frey, & Blencowe, 2008). The Human Genome Variation Society mandates that all variants can be described in relation to an accepted and clearly specified reference sequence and professional organizations’ guidelines recommend that alternate transcripts be considered in variant annotation (den Dunnen et al., 2016; Richards et al., 2015). However, at this time, commercial laboratories do not have standard guidelines for selecting a transcript for reporting, other than the longest or most clinically relevant transcript (Richards et al., 2015). In the case of Participant 10, the commercial laboratory reported the *KARS1* variants utilizing a transcript that was different from the one used in the literature. If comparisons of the participant's variants to the literature had only been made at the cDNA or protein level, without attention to the alternate transcripts, the inference about pathogenicity may have been overlooked. Publically available, web‐based and user‐friendly tools such as Ensembl Variant Effect Predictor and Mutalyzer allow users to generate corresponding variants in other transcripts (McLaren et al., 2010; Wildeman, van Ophuizen, den Dunnen, & Taschner, 2008), but the time and effort required may not be tractable in regular clinical settings.

#### 4.3.2 Phenotype‐driven reexamination of genomic data

ES identifies as many as 84,000 SNVs per individual (Belkadi et al., 2015), prohibiting manual consideration of each variant. Laboratories utilize various quality and control data as well as phenotype to filter detected variants to a short list for confirmation and reporting, and during this process may discard putative pathogenic variants (Eilbeck, Quinlan, & Yandell, 2017; Shamseldin et al., 2017). Illustrating this point is Participant 11, whose pathogenic *FBN1* variant was filtered out by the commercial laboratory due to phenotype mismatch and only found on manual inspection. While clinicians typically do not have direct access to the NGS data, if a diagnosis is highly suspected based on phenotype, it is possible to communicate with the laboratory to ask about coverage and the presence of rare and putatively deleterious variants in specific genes of interest. Thus, iterative interaction between the clinical and NGS analysis teams could enhance the probability of identifying the correct diagnosis.

#### 4.3.3 Exome reanalysis

Reanalyzing exome data can result in diagnosis due to interim bioinformatics improvements and newly reported gene‐disease associations, and is a practice supported by the ACMG (Deignan et al., 2019). Two large commercial ES laboratories recently reported that ES reanalysis of large patient cohorts resulted in a diagnostic yield of 10 to 23% (Guillen Sacoto et al., 2019, ASHG, abstract) (Liu et al., 2019). As part of a participant's UDN evaluation, ES data may be obtained from commercial laboratories for research‐based reanalysis, which at one clinical site resulted in diagnosis for 23% of participants with nondiagnostic pre‐UDN ES (Shashi et al., 2019). In the case of Participant 9, ES reanalysis resulted in a diagnosis of Ververi‐Brady syndrome due to recognition of the newly reported gene‐disease association (Ververi et al., 2018). In fact, approximately 250 new gene‐disease associations are curated by Online Mendelian Inheritance in Man (OMIM) each year (Wenger, Guturu, Bernstein, & Bejerano, 2017), which contribute to the majority of cases solved by reanalysis in clinical practice (Guillen Sacoto et al., 2019).

#### 4.3.4 Exome capture

Exome capture kits have improved since their initial development, allowing for better coverage (Shigemizu et al., 2015). One commercial ES laboratory estimates that the currently used capture kits interrogate roughly 3% more of the exome than the original version. This translates to approximately 1% of exome “negative” individuals from the earliest version (Agilent SureSelect Human All Exon V4) getting a diagnosis on the most recent version (IDT xGen exome). For example, if exome coverage was originally 95% and the diagnostic rate was 30%, then increasing coverage to 98% would increase the positive rate on the same cohort from 30% to 31% (K. Retterer, personal communication). Participant 12 demonstrates how limitations in exome capture can result in missed diagnosis, as the region of *SYNGAP1* containing the variant was not captured by the platform utilized by the commercial laboratory at that time. Notably, *SYNGAP1* is included in many intellectual disability/autism/epilepsy gene panels, which would likely have detected this variant, as coverage of exonic regions tends to be more complete on gene panels than on ES (Consugar et al., 2015; Xue, Ankala, Wilcox, & Hegde, 2015); and thus a gene panel may be better when a specific group of disorders are suspected.

## 5 CONCLUSIONS

The 12 participants described here illustrate how careful utilization of existing clinical resources with an individualized approach can provide answers for a subset of undiagnosed individuals. A thorough, personalized approach requires increased provider time commitment and sometimes additional financial resources, which can be challenging in clinical practice. A clear benefit of the UDN is the resources and dedicated time that are available for accepted participants that permit personalized and detailed attention to each case, resulting in highly likely or certain diagnoses for participants whose conditions had been refractory to prior diagnostic attempts.

## CONFLICT OF INTEREST

The Department of Molecular and Human Genetics at Baylor College of Medicine receives revenue from clinical genetic testing conducted at Baylor Genetics Laboratories.

## AUTHOR CONTRIBUTIONS

HC conceptualized, planned, and drafted the manuscript. VS contributed to the design, writing and revision of the manuscript. RS, JAR, EB, RS, KS, EG, EM, KGG contributed to the writing and revision of the manuscript. JAS, SL, JPO, GC, LCB, JEP, JP, AR, JAP, JC, and JMA participated in data collection and revision of the manuscript. All authors gave final approval of the version to be published and agree to be accountable for all aspects of the work in ensuring that questions related to the accuracy or integrity of any part of the work are appropriately investigated and resolved.

## CONSORTIA

Members of the Undiagnosed Diseases Network include, Maria T. Acosta, Margaret Adam, David R. Adams, Pankaj B. Agrawal, Mercedes E. Alejandro, Justin Alvey, Laura Amendola, Ashley Andrews, Euan A. Ashley, Mahshid S. Azamian, Carlos A. Bacino, Guney Bademci, Eva Baker, Ashok Balasubramanyam, Dustin Baldridge, Jim Bale, Michael Bamshad, Deborah Barbouth, Gabriel F. Batzli, Pinar Bayrak‐Toydemir, Anita Beck, Alan H. Beggs, Edward Behrens, Gill Bejerano, Jimmy Bennet, Beverly Berg‐Rood, Raphael Bernier, Jonathan A. Bernstein, Gerard T. Berry, Anna Bican, Stephanie Bivona, Elizabeth Blue, John Bohnsack, Carsten Bonnenmann, Devon Bonner, Lorenzo Botto, Brenna Boyd, Lauren C. Briere, Elly Brokamp, Gabrielle Brown, Elizabeth A. Burke, Lindsay C. Burrage, Manish J. Butte, Peter Byers, William E. Byrd, John Carey, Olveen Carrasquillo, Ta Chen Peter Chang, Sirisak Chanprasert, Hsiao‐Tuan Chao, Gary D. Clark, Terra R. Coakley, Laurel A. Cobban, Joy D. Cogan, F. Sessions Cole, Heather A. Colley, Cynthia M. Cooper, Heidi Cope, William J. Craigen, Andrew B. Crouse, Michael Cunningham, Precilla D'Souza, Hongzheng Dai, Surendra Dasari, Mariska Davids, Jyoti G. Dayal, Matthew Deardorff, Esteban C. Dell'Angelica, Shweta U. Dhar, Katrina Dipple, Daniel Doherty, Naghmeh Dorrani, Emilie D. Douine, David D. Draper, Laura Duncan, Dawn Earl, David J. Eckstein, Lisa T. Emrick, Christine M. Eng, Cecilia Esteves, Tyra Estwick, Marni Falk, Liliana Fernandez, Carlos Ferreira, Elizabeth L. Fieg, Paul G. Fisher, Brent L. Fogel, Irman Forghani, Laure Fresard, William A. Gahl, Emily Glanton, Ian Glass, Rena A. Godfrey, Katie Golden‐Grant, Alica M. Goldman, David B. Goldstein, Alana Grajewski, Catherine A. Groden, Andrea L. Gropman, Irma Gutierrez, Sihoun Hahn, Rizwan Hamid, Neil A. Hanchard, Kelly Hassey, Nichole Hayes, Frances High, Anne Hing, Fuki M. Hisama, Ingrid A. Holm, Jason Hom, Martha Horike‐Pyne, Alden Huang, Yong Huang, Rosario Isasi, Fariha Jamal, Gail P. Jarvik, Jeffrey Jarvik, Suman Jayadev, Jean M. Johnston, Lefkothea Karaviti, Jennifer Kennedy, Dana Kiley, Isaac S. Kohane, Jennefer N. Kohler, Deborah Krakow, Donna M. Krasnewich, Elijah Kravets, Susan Korrick, Mary Koziura, Joel B. Krier, Seema R. Lalani, Byron Lam, Christina Lam, Brendan C. Lanpher, Ian R. Lanza, C. Christopher Lau, Kimberly LeBlanc, Brendan H. Lee, Hane Lee, Roy Levitt, Richard A. Lewis, Sharyn A. Lincoln, Pengfei Liu, Xue Zhong Liu, Nicola Longo, Sandra K. Loo, Joseph Loscalzo, Richard L. Maas, Ellen F. Macnamara, Calum A. MacRae, Valerie V. Maduro, Marta M. Majcherska, May Christine V. Malicdan, Laura A. Mamounas, Teri A. Manolio, Rong Mao, Kenneth Maravilla, Thomas C. Markello, Ronit Marom, Gabor Marth, Beth A. Martin, Martin G. Martin, Julian A. Martínez‐Agosto, Shruti Marwaha, Jacob McCauley, Allyn McConkie‐Rosell, Colleen E. McCormack, Alexa T. McCray, Elisabeth McGee, Heather Mefford, J. Lawrence Merritt, Matthew Might, Ghayda Mirzaa, Eva Morava‐Kozicz, Paolo M. Moretti, Marie Morimoto, John J. Mulvihill, David R. Murdock, Mariko Nakano‐Okuno, Avi Nath, Stan F. Nelson, John H. Newman, Sarah K. Nicholas, Deborah Nickerson, Donna Novacic, Devin Oglesbee, James P. Orengo, Laura Pace, Stephen Pak, J. Carl Pallais, Christina GS. Palmer, Jeanette C. Papp, Neil H. Parker, John A. Phillips III, Jennifer E. Posey, Lorraine Potocki, Barbara N. Pusey, Aaron Quinlan, Wendy Raskind, Archana N. Raja, Genecee Renteria, Chloe M. Reuter, Lynette Rives, Amy K. Robertson, Lance H. Rodan, Jill A. Rosenfeld, Natalie Rosenwasser, Robb K. Rowley, Maura Ruzhnikov, Ralph Sacco, Jacinda B. Sampson, Susan L. Samson, Mario Saporta, C. Ron Scott, Judy Schaechter, Timothy Schedl, Kelly Schoch, Daryl A. Scott, Prashant Sharma, Vandana Shashi, Jimann Shin, Rebecca Signer, Catherine H. Sillari, Edwin K. Silverman, Janet S. Sinsheimer, Kathy Sisco, Edward C. Smith, Kevin S. Smith, Emily Solem, Lilianna Solnica‐Krezel, Rebecca C. Spillmann, Joan M. Stoler, Nicholas Stong, Jennifer A. Sullivan, Kathleen Sullivan, Angela Sun, Shirley Sutton, David A. Sweetser, Virginia Sybert, Holly K. Tabor, Cecelia P. Tamburro, Queenie K.‐G. Tan, Mustafa Tekin, Fred Telischi, Willa Thorson, Cynthia J. Tifft, Camilo Toro, Alyssa A. Tran, Brianna M. Tucker, Tiina K. Urv, Adeline Vanderver, Matt Velinder, Dave Viskochil, Tiphanie P. Vogel, Colleen E. Wahl, Stephanie Wallace, Nicole M. Walley, Chris A. Walsh, Melissa Walker, Jennifer Wambach, Jijun Wan, Lee‐kai Wang, Michael F. Wangler, Patricia A. Ward, Daniel Wegner, Mark Wener, Tara Wenger, Katherine Wesseling Perry, Monte Westerfield, Matthew T. Wheeler, Jordan Whitlock, Lynne A. Wolfe, Jeremy D. Woods, Shinya Yamamoto, John Yang, Guoyun Yu, Diane B. Zastrow, Chunli Zhao, Stephan Zuchner.

## WEB RESOURCES

Ensembl Variant Effect Predictor, https://useast.ensembl.org/info/docs/tools/vep/index.html

Mutalyzer, https://mutalyzer.nl/

RefGene, http://varianttools.sourceforge.net/Annotation/RefGene

Ensembl, https://useast.ensembl.org/index.html

UCSC Genome Browser, https://genome.ucsc.edu/

OMIM, https://omim.org/

## Data Availability

For all UDN research, specific variants are submitted to ClinVar (www.ncbi.nlm.gov/clinvar) and raw sequencing files are submitted to dbGAP (www.ncbi.nlm.hiv.gov/gap/) by the UDN Coordinating Center for UDN protocol, and therefore, are publically available.

## References

[mgg31397-bib-0001] Alfares, A. , Aloraini, T. , Subaie, L. A. , Alissa, A. , Qudsi, A. A. , Alahmad, A. , … Alfadhel, M. (2018). Whole‐genome sequencing offers additional but limited clinical utility compared with reanalysis of whole‐exome sequencing. Genetics in Medicine, 20(11), 1328–1333. 10.1038/gim.2018.41 29565419

[mgg31397-bib-0002] Attard, C. A. , Carmany, E. P. , & Trepanier, A. M. (2019). Genetic counselor workflow study: The times are they a‐changin'? J Genet Couns, 28(1), 130–140. 10.1002/jgc4.1041 30629774

[mgg31397-bib-0003] Ballif, B. C. , Theisen, A. , Coppinger, J. , Gowans, G. C. , Hersh, J. H. , Madan‐Khetarpal, S. , … Shaffer, L. G. (2008). Expanding the clinical phenotype of the 3q29 microdeletion syndrome and characterization of the reciprocal microduplication. Molecular Cytogenetics, 1, 8. 10.1186/1755-8166-1-8 18471269 PMC2408925

[mgg31397-bib-0004] Belkadi, A. , Bolze, A. , Itan, Y. , Cobat, A. , Vincent, Q. B. , Antipenko, A. , … Abel, L. (2015). Whole‐genome sequencing is more powerful than whole‐exome sequencing for detecting exome variants. Proceedings of the National Academy of Sciences of the United States of America, 112(17), 5473–5478. 10.1073/pnas.1418631112 25827230 PMC4418901

[mgg31397-bib-0005] Bick, D. , Jones, M. , Taylor, S. L. , Taft, R. J. , & Belmont, J. (2019). Case for genome sequencing in infants and children with rare, undiagnosed or genetic diseases. Journal of Medical Genetics, 56(12), 783–791. 10.1136/jmedgenet-2019-106111 31023718 PMC6929710

[mgg31397-bib-0006] Cao, Y. , Tokita, M. J. , Chen, E. S. , Ghosh, R. , Chen, T. , Feng, Y. , … Stankiewicz, P. (2019). A clinical survey of mosaic single nucleotide variants in disease‐causing genes detected by exome sequencing. Genome Medicine, 11(1), 48. 10.1186/s13073-019-0658-2 31349857 PMC6660700

[mgg31397-bib-0007] Carson, J. C. , Hoffner, L. , Conlin, L. , Parks, W. T. , Fisher, R. A. , Spinner, N. , … Surti, U. (2018). Diploid/triploid mixoploidy: A consequence of asymmetric zygotic segregation of parental genomes. American Journal of Medical Genetics. Part A, 176(12), 2720–2732. 10.1002/ajmg.a.40646 30302900

[mgg31397-bib-0008] Chen, X. , Schulz‐Trieglaff, O. , Shaw, R. , Barnes, B. , Schlesinger, F. , Kallberg, M. , … Saunders, C. T. (2016). Manta: Rapid detection of structural variants and indels for germline and cancer sequencing applications. Bioinformatics, 32(8), 1220–1222. 10.1093/bioinformatics/btv710 26647377

[mgg31397-bib-0009] Collod‐Beroud, G. , & Boileau, C. (2002). Marfan syndrome in the third Millennium. European Journal of Human Genetics, 10(11), 673–681. 10.1038/sj.ejhg.5200876 12404097 PMC2695985

[mgg31397-bib-0010] Consugar, M. B. , Navarro‐Gomez, D. , Place, E. M. , Bujakowska, K. M. , Sousa, M. E. , Fonseca‐Kelly, Z. D. , … Pierce, E. A. (2015). Panel‐based genetic diagnostic testing for inherited eye diseases is highly accurate and reproducible, and more sensitive for variant detection, than exome sequencing. Genetics in Medicine, 17(4), 253–261. 10.1038/gim.2014.172 25412400 PMC4572572

[mgg31397-bib-0011] Deignan, J. L. , Chung, W. K. , Kearney, H. M. , Monaghan, K. G. , Rehder, C. W. , Chao, E. C. , & Committee, A. L. Q. A. (2019). Points to consider in the reevaluation and reanalysis of genomic test results: A statement of the American College of Medical Genetics and Genomics (ACMG). Genetics in Medicine, 21(6), 1267–1270. 10.1038/s41436-019-0478-1 31015575 PMC6559819

[mgg31397-bib-0012] den Dunnen, J. T. , Dalgleish, R. , Maglott, D. R. , Hart, R. K. , Greenblatt, M. S. , McGowan‐Jordan, J. , … Taschner, P. E. (2016). HGVS Recommendations for the Description of Sequence Variants: 2016 Update. Human Mutation, 37(6), 564–569. 10.1002/humu.22981 26931183

[mgg31397-bib-0013] Eilbeck, K. , Quinlan, A. , & Yandell, M. (2017). Settling the score: Variant prioritization and Mendelian disease. Nature Reviews Genetics, 18(10), 599–612. 10.1038/nrg.2017.52 PMC5935497 28804138

[mgg31397-bib-0014] Fennell, A. P. , Hunter, M. F. , & Corboy, G. P. (2020). The changing face of clinical genetics service delivery in the era of genomics: A framework for monitoring service delivery and data from a comprehensive metropolitan general genetics service. Genetics in Medicine, 22(1), 210–218. 10.1038/s41436-019-0602-2 31292527

[mgg31397-bib-0015] Gahl, W. A. , Wise, A. L. , & Ashley, E. A. (2015). The undiagnosed diseases network of the National Institutes of Health: A national extension. JAMA, 314(17), 1797–1798. 10.1001/jama.2015.12249 26375289

[mgg31397-bib-0016] Gross, A. M. , Ajay, S. S. , Rajan, V. , Brown, C. , Bluske, K. , Burns, N. J. , … Taft, R. J. (2019). Copy‐number variants in clinical genome sequencing: Deployment and interpretation for rare and undiagnosed disease. Genetics in Medicine, 21(5), 1121–1130. 10.1038/s41436-018-0295-y 30293986 PMC6752263

[mgg31397-bib-0017] Guillen Sacoto, M. , Begtrup, A. , Willaert, R. , Crunk, A. , Heise, E. , Rhodes, L. , … Juusola, J. (2019). Outcomes for reanalysis of exome sequencing data in a large cohort. Paper presented at the American Society for Human Genetics Annual Meeting, Houston, TX.

[mgg31397-bib-0018] Hensel, C. , Vanzo, R. , Martin, M. , Dixon, S. , Lambert, C. , Levy, B. , … Wassman, E. (2017). Analytical and clinical validity study of FirstStepDx PLUS: A chromosomal microarray optimized for patients with neurodevelopmental conditions. PLoS Currents, 9. 10.1371/currents.eogt.7d92ce775800ef3fbc72e3840fb1bc22 PMC5346028 28357155

[mgg31397-bib-0019] Hoekstra, A. S. , van den Ende, B. , Julia, X. P. , van Breemen, L. , Scheurwater, K. , Tops, C. M. , … Bayley, J. P. (2017). Simple and rapid characterization of novel large germline deletions in SDHB, SDHC and SDHD‐related paraganglioma. Clinical Genetics, 91(4), 536–544. 10.1111/cge.12843 27485256

[mgg31397-bib-0020] Joshi, C. , Kolbe, D. L. , Mansilla, M. A. , Mason, S. O. , Smith, R. J. , & Campbell, C. A. (2016). Reducing the cost of the diagnostic odyssey in early onset epileptic encephalopathies. BioMed Research International, 2016, 6421039. 10.1155/2016/6421039 27243033 PMC4875968

[mgg31397-bib-0021] Karppa, M. , Kytovuori, L. , Saari, M. , & Majamaa, K. (2018). Mutation m.15923A>G in the MT‐TT gene causes mild myopathy ‐ Case report of an adult‐onset phenotype. BMC Neurology, 18(1), 149. 10.1186/s12883-018-1159-4 30236074 PMC6147040

[mgg31397-bib-0022] Kinsler, V. A. , Boccara, O. , Fraitag, S. , Torrelo, A. , Vabres, P. , & Diociaiuti, A. (2020). Mosaic abnormalities of the skin: Review and guidelines from the European Reference Network for rare skin diseases. British Journal of Dermatology, 182(3), 552–563. 10.1111/bjd.17924 30920652

[mgg31397-bib-0023] Korenke, G. C. , Krasemann, E. , Meier, V. , Beuche, W. , Hunneman, D. H. , & Hanefeld, F. (1998). First missense mutation (W679R) in exon 10 of the adrenoleukodystrophy gene in siblings with adrenomyeloneuropathy. Human Mutation, 11(S1), S204–S206. 10.1002/humu.1380110166 9452087

[mgg31397-bib-0024] Krumm, N. , Sudmant, P. H. , Ko, A. , O'Roak, B. J. , Malig, M. , Coe, B. P. , … Eichler, E. E. (2012). Copy number variation detection and genotyping from exome sequence data. Genome Research, 22(8), 1525–1532. 10.1101/gr.138115.112 22585873 PMC3409265

[mgg31397-bib-0025] Liu, P. , Meng, L. , Normand, E. A. , Xia, F. , Song, X. , Ghazi, A. , … Yang, Y. (2019). Reanalysis of clinical exome sequencing data. New England Journal of Medicine, 380(25), 2478–2480. 10.1056/NEJMc1812033 31216405 PMC6934160

[mgg31397-bib-0026] Maiese, D. R.., Keehn, A. , Lyon, M. , Flannery, D. , & Watson, M. ; Working Groups of the National Coordinating Center for Seven Regional Genetics Service, C . (2019). Current conditions in medical genetics practice. Genetics in Medicine, 21(8), 1874–1877. 10.1038/s41436-018-0417-6 30686822 PMC6752678

[mgg31397-bib-0027] Manning, M. , Hudgins, L. , Professional, P. , & Guidelines, C. (2010). Array‐based technology and recommendations for utilization in medical genetics practice for detection of chromosomal abnormalities. Genetics in Medicine, 12(11), 742–745. 10.1097/GIM.0b013e3181f8baad 20962661 PMC3111046

[mgg31397-bib-0028] Mason‐Suares, H. , Kim, W. , Grimmett, L. , Williams, E. S. , Horner, V. L. , Kunig, D. , … Rudd, M. K. (2013). Density matters: Comparison of array platforms for detection of copy‐number variation and copy‐neutral abnormalities. Genetics in Medicine, 15(9), 706–712. 10.1038/gim.2013.36 23558256

[mgg31397-bib-0029] McLaren, W. , Pritchard, B. , Rios, D. , Chen, Y. , Flicek, P. , & Cunningham, F. (2010). Deriving the consequences of genomic variants with the Ensembl API and SNP Effect Predictor. Bioinformatics, 26(16), 2069–2070. 10.1093/bioinformatics/btq330 20562413 PMC2916720

[mgg31397-bib-0030] McMillan, H. J. , Humphreys, P. , Smith, A. , Schwartzentruber, J. , Chakraborty, P. , Bulman, D. E. , … Geraghty, M. T. (2015). Congenital visual impairment and progressive microcephaly due to lysyl‐transfer ribonucleic acid (RNA) synthetase (KARS) mutations: The expanding phenotype of aminoacyl‐transfer rna synthetase mutations in human disease. Journal of Child Neurology, 30(8), 1037–1043. 10.1177/0883073814553272 25330800

[mgg31397-bib-0031] Miller, D. T. , Adam, M. P. , Aradhya, S. , Biesecker, L. G. , Brothman, A. R. , Carter, N. P. , … Ledbetter, D. H. (2010). Consensus statement: Chromosomal microarray is a first‐tier clinical diagnostic test for individuals with developmental disabilities or congenital anomalies. American Journal of Human Genetics, 86(5), 749–764. 10.1016/j.ajhg.2010.04.006 20466091 PMC2869000

[mgg31397-bib-0032] Mirzaa, G. , Timms, A. E. , Conti, V. , Boyle, E. A. , Girisha, K. M. , Martin, B. , Dobyns, W. B. (2016). PIK3CA‐associated developmental disorders exhibit distinct classes of mutations with variable expression and tissue distribution. JCI Insight, 1(9). 10.1172/jci.insight.87623 PMC5019182 27631024

[mgg31397-bib-0033] Neul, J. L. , Kaufmann, W. E. , Glaze, D. G. , Christodoulou, J. , Clarke, A. J. , Bahi‐Buisson, N. , … RettSearch, C. (2010). Rett syndrome: Revised diagnostic criteria and nomenclature. Annals of Neurology, 68(6), 944–950. 10.1002/ana.22124 21154482 PMC3058521

[mgg31397-bib-0034] Nguengang Wakap, S. , Lambert, D. M. , Olry, A. , Rodwell, C. , Gueydan, C. , Lanneau, V. , … Rath, A. (2019). Estimating cumulative point prevalence of rare diseases: Analysis of the Orphanet database. European Journal of Human Genetics, 10.1038/s41431-019-0508-0 PMC6974615 31527858

[mgg31397-bib-0035] Pan, Q. , Shai, O. , Lee, L. J. , Frey, B. J. , & Blencowe, B. J. (2008). Deep surveying of alternative splicing complexity in the human transcriptome by high‐throughput sequencing. Nature Genetics, 40(12), 1413–1415. 10.1038/ng.259 18978789

[mgg31397-bib-0036] Parikh, S. , Goldstein, A. , Koenig, M. K. , Scaglia, F. , Enns, G. M. , Saneto, R. , … DiMauro, S. (2015). Diagnosis and management of mitochondrial disease: A consensus statement from the Mitochondrial Medicine Society. Genetics in Medicine, 17(9), 689–701. 10.1038/gim.2014.177 25503498 PMC5000852

[mgg31397-bib-0037] Pena, L. D. M. , Jiang, Y. H. , Schoch, K. , Spillmann, R. C. , Walley, N. , Stong, N. , … Shashi, V. (2018). Looking beyond the exome: A phenotype‐first approach to molecular diagnostic resolution in rare and undiagnosed diseases. Genetics in Medicine, 20(4), 464–469. 10.1038/gim.2017.128 28914269 PMC5851806

[mgg31397-bib-0038] Posey, J. E. (2019). Genome sequencing and implications for rare disorders. Orphanet Journal of Rare Diseases, 14(1), 153. 10.1186/s13023-019-1127-0 31234920 PMC6591893

[mgg31397-bib-0039] Posey, J. E. , Rosenfeld, J. A. , James, R. A. , Bainbridge, M. , Niu, Z. , Wang, X. , … Plon, S. E. (2016). Molecular diagnostic experience of whole‐exome sequencing in adult patients. Genetics in Medicine, 18(7), 678–685. 10.1038/gim.2015.142 26633545 PMC4892996

[mgg31397-bib-0040] Ramoni, R. B. , Mulvihill, J. J. , Adams, D. R. , Allard, P. , Ashley, E. A. , Bernstein, J. A. , … Wise, A. L. (2017). The undiagnosed diseases network: Accelerating discovery about health and disease. American Journal of Human Genetics, 100(2), 185–192. 10.1016/j.ajhg.2017.01.006 28157539 PMC5294757

[mgg31397-bib-0041] Richards, S. , Aziz, N. , Bale, S. , Bick, D. , Das, S. , Gastier‐Foster, J. , … Committee, A. L. Q. A. (2015). Standards and guidelines for the interpretation of sequence variants: A joint consensus recommendation of the American College of Medical Genetics and Genomics and the Association for Molecular Pathology. Genetics in Medicine, 17(5), 405–424. 10.1038/gim.2015.30 25741868 PMC4544753

[mgg31397-bib-0042] Santibanez‐Koref, M. , Griffin, H. , Turnbull, D. M. , Chinnery, P. F. , Herbert, M. , & Hudson, G. (2019). Assessing mitochondrial heteroplasmy using next generation sequencing: A note of caution. Mitochondrion, 46, 302–306. 10.1016/j.mito.2018.08.003 30098421 PMC6509278

[mgg31397-bib-0043] Scocchia, A. , Wigby, K. M. , Masser‐Frye, D. , Del Campo, M. , Galarreta, C. I. , Thorpe, E. , … Taft, R. J. (2019). Clinical whole genome sequencing as a first‐tier test at a resource‐limited dysmorphology clinic in Mexico. NPJ Genomic Medicine, 4, 5. 10.1038/s41525-018-0076-1 30792901 PMC6375919

[mgg31397-bib-0044] Shamseldin, H. E. , Maddirevula, S. , Faqeih, E. , Ibrahim, N. , Hashem, M. , Shaheen, R. , & Alkuraya, F. S. (2017). Increasing the sensitivity of clinical exome sequencing through improved filtration strategy. Genetics in Medicine, 19(5), 593–598. 10.1038/gim.2016.155 27711071

[mgg31397-bib-0045] Shashi, V. , Schoch, K. , Spillmann, R. , Cope, H. , Tan, Q. K. , Walley, N. , … Goldstein, D. B. (2019). A comprehensive iterative approach is highly effective in diagnosing individuals who are exome negative. Genetics in Medicine, 21(1), 161–172. 10.1038/s41436-018-0044-2 29907797 PMC6295275

[mgg31397-bib-0046] Shigemizu, D. , Momozawa, Y. , Abe, T. , Morizono, T. , Boroevich, K. A. , Takata, S. , … Tsunoda, T. (2015). Performance comparison of four commercial human whole‐exome capture platforms. Scientific Reports, 5, 12742. 10.1038/srep12742 26235669 PMC4522667

[mgg31397-bib-0047] Smith, E. D. , Blanco, K. , Sajan, S. A. , Hunter, J. M. , Shinde, D. N. , Wayburn, B. , … Radtke, K. (2019). A retrospective review of multiple findings in diagnostic exome sequencing: Half are distinct and half are overlapping diagnoses. Genetics in Medicine, 21(10), 2199–2207. 10.1038/s41436-019-0477-2 30894705 PMC6774997

[mgg31397-bib-0048] Spillmann, R. C. , McConkie‐Rosell, A. , Pena, L. , Jiang, Y. H. , Undiagnosed Diseases, N. , Schoch, K. , … Shashi, V. (2017). A window into living with an undiagnosed disease: Illness narratives from the Undiagnosed Diseases Network. Orphanet Journal of Rare Diseases, 12(1), 71. 10.1186/s13023-017-0623-3 28416019 PMC5392939

[mgg31397-bib-0049] Splinter, K. , Adams, D. R. , Bacino, C. A. , Bellen, H. J. , Bernstein, J. A. , Cheatle‐Jarvela, A. M. , … Undiagnosed Diseases, N. (2018). Effect of genetic diagnosis on patients with previously undiagnosed disease. New England Journal of Medicine, 379(22), 2131–2139. 10.1056/NEJMoa1714458 30304647 PMC6481166

[mgg31397-bib-0050] Srivastava, S. , Love‐Nichols, J. A. , Dies, K. A. , Ledbetter, D. H. , Martin, C. L. , Chung, W. K. , … … Group, N. D. D. E. S. R. W. (2019). Meta‐analysis and multidisciplinary consensus statement: Exome sequencing is a first‐tier clinical diagnostic test for individuals with neurodevelopmental disorders. Genetics in Medicine, 21(11), 2413–2421. 10.1038/s41436-019-0554-6 31182824 PMC6831729

[mgg31397-bib-0051] Tattini, L. , D'Aurizio, R. , & Magi, A. (2015). Detection of genomic structural variants from next‐generation sequencing data. Frontiers in Bioengineering and Biotechnology, 3, 92. 10.3389/fbioe.2015.00092 26161383 PMC4479793

[mgg31397-bib-0052] Truty, R. , Paul, J. , Kennemer, M. , Lincoln, S. E. , Olivares, E. , Nussbaum, R. L. , & Aradhya, S. (2019). Prevalence and properties of intragenic copy‐number variation in Mendelian disease genes. Genetics in Medicine, 21(1), 114–123. 10.1038/s41436-018-0033-5 29895855 PMC6752305

[mgg31397-bib-0053] Ververi, A. , Splitt, M. , Dean, J. C. S. , Study, D. D. D. , & Brady, A. F. (2018). Phenotypic spectrum associated with de novo mutations in QRICH1 gene. Clinical Genetics, 93(2), 286–292. 10.1111/cge.13096 28692176

[mgg31397-bib-0054] Webb, T. , & Latif, F. (2001). Rett syndrome and the MECP2 gene. Journal of Medical Genetics, 38(4), 217–223. 10.1136/jmg.38.4.217 11283201 PMC1734858

[mgg31397-bib-0055] Wenger, A. M. , Guturu, H. , Bernstein, J. A. , & Bejerano, G. (2017). Systematic reanalysis of clinical exome data yields additional diagnoses: Implications for providers. Genetics in Medicine, 19(2), 209–214. 10.1038/gim.2016.88 27441994

[mgg31397-bib-0056] Wildeman, M. , van Ophuizen, E. , den Dunnen, J. T. , & Taschner, P. E. (2008). Improving sequence variant descriptions in mutation databases and literature using the Mutalyzer sequence variation nomenclature checker. Human Mutation, 29(1), 6–13. 10.1002/humu.20654 18000842

[mgg31397-bib-0057] Xue, Y. , Ankala, A. , Wilcox, W. R. , & Hegde, M. R. (2015). Solving the molecular diagnostic testing conundrum for Mendelian disorders in the era of next‐generation sequencing: Single‐gene, gene panel, or exome/genome sequencing. Genetics in Medicine, 17(6), 444–451. 10.1038/gim.2014.122 25232854

[mgg31397-bib-0058] Zanzottera, C. , Milani, D. , Alfei, E. , Rizzo, A. , D'Arrigo, S. , Esposito, S. , & Pantaleoni, C. (2017). ZC4H2 deletions can cause severe phenotype in female carriers. American Journal of Medical Genetics. Part A, 173(5), 1358–1363. 10.1002/ajmg.a.38155 28345801

[mgg31397-bib-0059] Zhou, X. L. , He, L. X. , Yu, L. J. , Wang, Y. , Wang, X. J. , Wang, E. D. , & Yang, T. (2017). Mutations in KARS cause early‐onset hearing loss and leukoencephalopathy: Potential pathogenic mechanism. Human Mutation, 38(12), 1740–1750. 10.1002/humu.23335 28887846

